# Moving and sensing without input and output: early nervous systems and the origins of the animal sensorimotor organization

**DOI:** 10.1007/s10539-015-9483-1

**Published:** 2015-03-03

**Authors:** Fred Keijzer

**Affiliations:** Department of Theoretical Philosophy, University of Groningen, Oude Boteringestraat 52, 9712 GL Groningen, The Netherlands

**Keywords:** Early nervous systems, Skin brain thesis, Sensorimotor organization, Embodiment, Input–output view, Coordination view

## Abstract

It remains a standing problem how and why the first nervous systems evolved. Molecular and genomic information is now rapidly accumulating but the macroscopic organization and functioning of early nervous systems remains unclear. To explore potential evolutionary options, a coordination centered view is discussed that diverges from a standard input–output view on early nervous systems. The scenario involved, the skin brain thesis (SBT), stresses the need to coordinate muscle-based motility at a very early stage. This paper addresses how this scenario with its focus on coordination also deals with sensory aspects. It will be argued that the neural structure required to coordinate extensive sheets of contractile tissue for motility provides the starting point for a new multicellular organized form of sensing. Moving a body by muscle contraction provides the basis for a multicellular organization that is sensitive to external surface structure at the scale of the animal body. Instead of thinking about early nervous systems as being connected to the environment merely through input and output, the implication developed here is that early nervous systems provide the foundation for a highly specific animal sensorimotor organization in which neural activity directly reflects bodily and environmental spatiotemporal structure. While the SBT diverges from the input–output view, it is closely linked to and supported by ongoing work on embodied approaches to intelligence to which it adds a new interpretation of animal embodiment and sensorimotor organization.

## Introduction

It remains a standing problem how and why the first nervous systems evolved. From the nineteenth century onward, scientists proposed various evolutionary scenarios but the issue was never resolved. Nowadays, molecular and in particular genomic data have made large inroads into the phylogenetic relations between different animal lineages but still no clear evolutionary sequence for the origins of the first nervous systems has emerged.^1^ Instead, it has become a debate whether neurons, nerve nets and central nervous systems evolved several times independently in separate lineages (Moroz 2009; Northcutt 2012).

An important way to make progress here is by improving our scenarios of how very early nervous systems may have functioned. Sophisticated scenario building should address the proximate and macroscopic operation of plausible early nervous system configurations as well as of protonervous systems that span the gap between nervous systems proper and other tissues. So far, almost all proposals targeted a too limited domain of functional options. Our appreciation of the functional options available for early nervous systems can be improved by drawing a contrast between two broad interpretations of primitive functioning. The first interpretation sees nervous systems primarily as connecting devices that link sensors to effectors. In this case, the simplest nervous system that is conceptually possible consists of a single neuron that connects a sensor to an effector (Parker 1919; Mackie 1990; Lichtneckert and Reichert 2007; Miller 2009, Jékely 2011). This interpretation that will be referred to as the *input*–*output view* is closely linked to the standard interpretation for modern nervous systems (e.g. Braitenberg 1984; Greenspan 2007). A contrasting interpretation, the *coordination view*, sees early nervous systems primarily as coordination devices enabling motility by increasingly large multicellular organisms (Pantin 1956; Passano 1963; Keijzer et al. 2013). Conceptually, the simplest nervous system possible here would be a diffuse nerve net consisting of a significant number of neurons spread out over a large portion of the animal’s body, such as can nowadays be seen in *Hydra* (Grimmelikhuijzen 1985). While both interpretations should be considered as purely hypothetical at present, much more attention has been given to the first, skewing research towards this interpretation, while disregarding the issues raised by the second interpretation.

Both an input–output view and a coordination view can take many different forms. The present paper will develop a particular coordination interpretation in some detail. The view developed here consists of the skin brain thesis^2^ or SBT (Keijzer et al. 2013). The SBT proposes an evolutionary progression from excitable and contractile epithelia to full nerve nets as increasingly sophisticated ways to coordinate contractile tissues such as muscle. However, as remarked by Monk (2014), the SBT as formulated earlier seems to stress only motility and to neglect sensory aspects that are obviously central to nervous system organization and evolution as well. Thus the specific aim of the present paper will be to sketch how the SBT deals with sensing as well as motility.

According to the SBT, the use of *tissue contraction* for motility was the initial key feature that provided both an evolutionarily new form of motility for early animals as well as an evolutionary new means to sense extended surface structures in the environment. In this view, tissue contraction provided the basis for a specific *animal* sensorimotor organization or ASMO. In this account, the evolution of nervous systems and contractile tissues are fully intertwined and together they turned the animal body into the motile and sensing unit that became so evolutionarily successful at the start of the Cambrian.

The ASMO is specific to animals and will be contrasted to the idea that an animal sensorimotor organization at heart similar to a robotic device equipped with various sensors and effectors (e.g. O’Regan and Noë 2001) as implied by the input–output view. The aim is not to criticize such an input–output view, but only to develop the SBT as a viable and attractive alternative view on early nervous system evolution. Obviously, by elaborating the soundness of the SBT while stressing the differences with an input–output view, the result may not be completely even-handed. However, critique is not the aim.

Formulating a coordination-based alternative for the dominant input–output view will expand the conceptual options that are available for making sense of early nervous system evolution. While having such an alternative is a good thing in itself by extending the range of options, it also provides competition that will force defenders of the dominant view to develop their own range of scenarios in more detail. At another level, the coordination view developed below connects more widely with cognitive and neuroscience theorizing. More specifically, the SBT and the ASMO notion are closely related to embodied approaches to cognition (e.g. Van Gelder 1995; Brooks 1999; Keijzer 2001; Pfeifer and Bongard 2007; Thompson 2007; Clark 2008; Chemero 2009; Barrett 2011) as well as sensorimotor approaches to cognition and consciousness (e.g. O’Regan 2011; O’Regan and Noë 2001; Hurley 2001; Chemero 2009; Buhrmann et al. 2013). This embodied and sensorimotor work provides a wider conceptual and empirical backing for the SBT, while the latter provides an outline how these embodied and sensorimotor approaches invite new conceptual and empirical interpretations of nervous systems more generally.

The text has the following structure. The next section introduces the Precambrian setting in which early nervous systems can be assumed to have evolved. The “The input–output view on nervous systems and the animal sensorimotor organization” section sketches the input–output view to which the coordination view will be contrasted. “The skin brain thesis: coordinating contraction-based motility” section sets out the main features of the skin brain thesis as the most recent version of a coordination view. “Pantin patterning instead of output” and “Sensing bodies rather than input” sections provide the argument for an *animal* sensorimotor organization, ASMO, by sketching respectively how the skin brain proposal provides the ingredients for self-induced motility that is not output, and for a form of sensing that is not input. The concluding section will highlight the main features of the ASMO by contrasting them with those of the input–output view.

## The Precambrian roots of nervous systems and the animal sensorimotor organization

What can we say about the context in which the first nervous systems evolved? A first port of call is the famous Cambrian explosion, a comparatively short period lasting from about 530 to 515 Ma (million years ago) during which most of the modern animal phyla first appeared in a recognizable form in the fossil record (Valentine 2004). From here on ‘standard’ animals with brains, multiple appendages, eyes, heads, tails and hard skeletons roamed the Earth. Many innovations in morphology and behavior first appeared during this period, and these could very well have driven the ‘explosion’. For example, Andrew Parker’s (2003) ‘light switch’ theory takes the evolution of the first eyes as the key change that initiated a new predatory life-style for arthropods. This idea can be combined with prey-predator co-evolution, which is indicated by the evolution of shells, burrowing and hard mouth parts (e.g. Vermeij 1989; Conway Morris and Bengtson 1994; Dzik 2007). Ginsburg and Jablonka (2010) suggest that these prey-predator interactions were driven by associative learning together with a centralization of nervous systems, while Trestman (2013) stresses that complex cognitive embodiment enabled tracking the spatial properties and relations within the environment. In all cases, increasingly sophisticated behavior is cast as a key reason behind the Cambrian explosion.

However, all these proposals require the presence of nervous systems and this implies that their evolution must have taken place earlier (e.g. Fedonkin et al. 2007; Brasier 2009; Erwin et al. 2011; Zhang et al. 2014). The lower boundary of the Cambrian itself (541 Ma) is marked by the presence of the trace fossil *Treptichnus pedum* (Erwin et al. 2011; Landing 1994) which consists of burrowing traces similar to those of modern priapulid worms (Vannier et al. 2010). Preceding periods lack such bioturbation of the sea floor, making the presence of active burrowing animals before the Cambrian unlikely (Fedonkin et al. 2007; Brasier 2009). As active burrowing requires a hydrostatic skeleton, muscle tissue and a nervous system, the logical conclusion is that nervous systems originated before the Cambrian, either in the Ediacaran (635–541 Ma) or even the Cryogenian (850–635 Ma).

Current knowledge about animal life during the Precambrian period is rather tentative. It derives from various sources that do not all converge nicely. First, macroscopic Ediacaran body fossils have been found that are dated from 579 to 541 Ma. Most are difficult to relate to modern animal phyla or to interpret behaviorally. For example, there is an ongoing debate whether the emblematic *Dickinsonia* did (Gehling et al. 2005) or did not (Brasier and Antcliffe 2008) actively graze and change its position on the substrate, and also whether they can be presumed to have some form of nervous system. In addition Ediacaran trace fossils are found (Droser et al. 2005). Unquestioned examples of millimeter wide surface traces go back to at least 560 Ma (Jensen et al. 2006; Liu et al. 2010). These traces suggest that these organisms remained very small and worm-like, possibly moving by ciliary creeping rather than muscle (Valentine 2007) and possibly without a nervous system until late in the Ediacaran.

Molecular data add to this picture. A lot of the important genetic and molecular ingredients for animal life were already present at a very early stage, often going back to the last common shared ancestor, single celled choanoflagellates (King 2004). For example, key components of the postsynaptic density—itself an important part of the mammalian synapse—were already present in sponges (Sakarya et al. 2007) and some of them even in fungi and choanoflagellates (Ryan and Grant 2009). A similar story can be told for the evolution of sodium channels, also present in choanoflagellates (Liebeskind et al. 2011). In addition, the gene repertoire and genomic organization of cnidarians—such as jellyfish and sea anemones—is already remarkably complex and matches many human sequences (Putnam et al. 2007; Technau et al. 2005), while extensive gene loss can be observed in classic model organisms such as *Drosophila* and *Caenorhabditis* (Kortschak et al. 2003). This ancestral genetic complexity also applies to neurodevelopment as cnidarians possess an almost complete set of the signaling molecules that have a critical role in bilaterian neurodevelopment (Watanabe et al. 2009). Together, these molecular data suggest significant morphological complexity at an early stage, although the morphology itself remains unknown (Erwin 2005).

Work on molecular clocks aims to position these molecular changes and inventions in time by setting dates for the splits between animal lineages. However, the dates arrived at by these molecular clocks are very early compared to the fossil record (e.g. Blair 2009), despite efforts at calibrating the two (Peterson et al. 2008; Erwin et al. 2011). For example, the deep divergences between animal lineages are set in the Cryogenian, thus before 635 Ma. As some of the split lineages already share the presence of nervous systems, this suggests that nervous systems evolved very early and were present in different animal lineages for tens of millions of years without leaving any fossil evidence, presumably because they remained very small (e.g. Brasier 2009). Alternatively, one may reject these implications of the molecular data and stick to the fossil evidence that positions the relevant evolutionary changes right at the Ediacaran-Cambrian boundary (Monk and Paulin 2014). One way to do this is to accept that the important lineages split very early, but also argue that nervous systems evolved much later and independently in different lineages (Moroz 2009, 2014; Northcutt 2012). Without additional confirmation for any of these options, the absolute timing of early nervous systems evolution should be considered as contested. This wide time range makes it also difficult to be specific about the ecological conditions and the specific selective environment under which nervous system evolution occurred: ecological conditions, such as global temperature and oxygen levels, changed dramatically during the Proterozoic (Gaidos et al. 2007).

Thus, while our knowledge at the molecular level has vastly increased, much less is known about the macroscopic functioning and organization of the organisms that first evolved nervous systems, or about the ecological circumstances under which they lived. Given the scarcity of tangible evidence, specifying schemes that sketch how a non-neural organization could have been transformed into a nervous system would help to further constrain research on early nervous systems.

Both the input–output view and the coordination view provide scenarios for this transition. Many of these proposals do not involve highly specific adaptive constraints. Instead, these scenarios can be better interpreted as *lineage explanations* (Calcott 2009). Such explanations specify a series of phenotypic stages; each stage shows how a particular phenotypic organization acts as a mechanism that fulfils some particular task; the differences between adjacent stages demonstrate how these mechanisms could change into the next stage’s mechanism through minor modifications. Nilsson and Pelger’s evolutionary sequence of eye-organizations that increasingly enhance visual acuity constitutes a classic example of such a sequence (ibid.). Given their proximate focus, neither the currently unknown adaptive conditions under which these mechanisms functioned, nor the particular animal in which they were embedded need to be addressed by such explanations.

Lineage explanations for the evolution of early nervous systems thus involve specifying a sequence of neural organizations, all acting as a mechanism that performs a plausible adaptive task. While both the input–output view and the coordination view propose such sequences, they differ in their take on what counts as the plausible task that early neural organizations performed to enable a motile life-style. The dominant input–output view interprets nervous systems foremost as a mechanism that allowed organisms to react (more) adequately to the environment by connecting sensors and effectors. In contrast, the lesser known coordination view stresses the initial need to organize bodily coordination as the main purpose. This difference in view on what nervous systems initially were for has important implications for the scenarios that are built on these views.

## The input–output view on nervous systems and the animal sensorimotor organization

Developing scenarios for early nervous system evolution has a long history. Most of them fit within an input–output view. To position the contrasting coordination view developed here, a short sketch of what is typical for the input–output view will be helpful. The input–output view is an idealized general interpretation of nervous systems’ functioning that also provides the background for most proposals for the origins of nervous systems. Two issues must be stressed. First, it is important to differentiate the wider use of input–output terminology from the specific view on nervous systems and their evolution. Only the latter will be discussed here. Second, the input–output view is so generally accepted that it is usually only implicit. For example, the idea that brains process information is so widespread within psychology, cognitive- and neuroscience that this particular claim is hardly ever argued for but just assumed as given. In the following, I aim to explicate some of the most typical and relevant characteristics of the input–output view to provide a contrast with a coordination view.

According to the input–output view, nervous systems are at heart information processing input–output devices: nervous systems receive information through sensors, process this information, and subsequently use this information to instruct and modulate effectors. In basic forms, this is envisioned as an almost direct pathway as exemplified by the classic spinal reflex. In more complex cases, a central brain intermediates but the input–output view for whole nervous systems remains in place as brains are conceived of as way stations between afferent and efferent fibers that connect it to sensors and effectors. Feedback and reafference relations add to the basic picture but do not change it fundamentally. The view on nervous systems is closely tied to an input–output view on animal embodiment and its sensorimotor organization. Under the input–output view this sensorimotor organization consists of the sensor and effector capabilities of animals that connect the nervous system to the world. The animal body constitutes a platform that puts the senses and effectors in specific configurations that enable and constrain the specific sensorimotor interactions any animal is capable of (e.g. Clark 2008).

Most scenarios for early nervous system evolution start from here and aim to provide an account how nervous systems came to be interposed between a set of effectors and a set of sensors. For example, George Parker (1919) made the still influential proposal for an evolutionary sequence of (a) independent effectors, (b) direct sensor-effector systems, and (c) a sensory-interneuron-effector organization. Since then there have been many variations on this basic theme (Lichtneckert and Reichert 2007) with Jékely’s (2011) as an ingenious recent version. These proposals are well reviewed (see footnote 1) and will not be discussed here. Instead it will be useful to specify some of the central characteristics of the input–output view.

An iconic illustration of these characteristics is provided by Braitenberg’s sequence of hypothetical artificial vehicles (Braitenberg 1984). These vehicles are small carts with sensors at the front and one or more motors driving it forward. Braitenberg envisioned a systematic progression of these vehicles to illuminate the evolutionary change towards more complex nervous systems and eventually the human brain. The most basic nervous system that is conceptually possible within this view consists of a single sensor, a single motor and a single neural connection between the two. This basic configuration is extended in a quasi-evolutionary way by providing more extensive sensor arrays, more complex effectors and more complex wiring systems between the two. This enabled increasingly complex behavior and complex sensitivity to environmental features.

The image of Braitenberg’s vehicles highlights a number of typical features of the input–output view on nervous systems and the sensorimotor organization to which it is connected. The following list specifies some of these features, selected here with an eye to provide specific points of contrast for the coordination view developed below.

1. Animal- and current artificial sensorimotor organizations operate similarly;
2. sensors and effectors are prior to nervous systems;
3. a direct connection between a sensor and an effector is the most basic form nervous systems can take;
4. the resulting sensorimotor system is mostly passive and put into motion by external stimuli;
5. activity within nervous systems is primarily through-conducting, traveling from sensors to effectors;
6. nervous systems process environmental information;
7. the animal body constitutes a stable platform for sensors and effectors, the precise placement of which is central to behavior;
8. the animal body houses a nervous system but the latter remains separate, except through its sensors and effectors.

All these features follow directly from the imagery provided by Braitenberg’s artificial vehicles: the similarity of the artificial and the biological; the priority of sensors and effectors; the minimal case cast as a direct connection between them; the inherent passivity and through-conducting organization and so on. The relevance of some of them—in particular 7 and 8—will only become apparent after discussing the alternative.

These features are meant to be typical and conceptually influential rather than a rigid set of necessary conditions that specify the input–output view. For example, the feature of passivity may be questioned given the central role of internal pacemakers (e.g. Selverston 2010) or the observation that it is not a necessary requirement. Nevertheless, passivity remains an influential background assumption in large parts of neuroscience where reactions to stimuli are held central rather than endogenous activity (Raichle 2010) and thus can be considered typical. Finally, this sketch of the input–output view and the following comparison is not to criticize this view. The aim is to articulate some of its central features in order to show in what way the skin brain thesis, as a coordination view, differs on these features, and therefore how it adds to the conceptual options available for understanding nervous systems and their evolution (Jékely et al. in preparation).

## The skin brain thesis: coordinating contraction-based motility

The skin brain thesis (SBT) provides a general outline how early nervous systems could have evolved as organs that coordinate contraction-based motility (Keijzer et al. 2013). It sketches how and why a multicellular organization evolved that turned clusters of cells into integrated behavioral units. This section provides a short overview of the main ideas.

The SBT builds on Carl Pantin’s proposal that the earliest nervous systems evolved to coordinate what he called the animal ‘behavior machine’ (1952, 1956). With this phrase he referred to the way in which animals relied on muscle and other contractile tissues such as myoepithelia to generate motility. The SBT explicitly takes contraction-based motility as the kernel of nervous system organization and of the ASMO. This may not seem obvious as many animals have other means of motility, most notably the use of cilia: cellular extensions that can act like small ‘oars’ propelling the organism forward.^3^ Nevertheless, contractile tissue is definitely the most typical, powerful and dominant source of animal motility. Without it the large motile animals of the Cambrian and onward could not exist as motility by cilia comes with strong size and efficiency constraints that are only overcome by contractile tissue (Seipel and Schmid 2005). Muscle forms the animal’s ‘prime mover’ (Vogel 2003) and is the key feature of the standard sensorimotor organization of modern animals, constituting around 40 % of human body weight. The rise of muscle-based motility is unquestionably a key transition in animal evolution. The SBT proposes that contraction-based motility goes back to the very origins of nervous systems and that both are intrinsically related. The main claim is that this source of animal motility is more than an essentially arbitrary effector, contraction-based motility is central to nervous systems and the ASMO itself.

Following Pantin, the SBT stresses that contraction-based motility requires the integration of such contractions across the whole—or at least large parts of—the animal’s body. The SBT also emphasizes that an account of early nervous systems should derive from generic features that can be assumed to have been present in a Precambrian context and that could be utilized for this new purpose of coordination, such as the presence of electrically excitable cells, chemical signaling between cells and contractile cells. It also assumes an epithelial organization that, together with the extracellular matrix it creates, constitutes the fundamental building material of animals making up all their intricate forms and internal spaces (Holland 2011; Leys and Riesgo 2011). Like Pantin, the SBT stresses spontaneous activity within the organism, such as internal pacemakers (Passano 1963; Lichtneckert and Reichert 2007). Pantin himself acknowledged the presence of direct reflexes to specific stimuli, but thought of these as secondary developments that could only evolve and exist in the context of a whole acting organism, a position that is followed by the SBT. In all cases, the need for a functional understanding of how an elementary contraction-based motility mechanism might have worked and evolved is central.

At a neuronal level, the SBT differentiates between two essential properties of modern neurons: (a) Neurons have synapses that enable electrical signaling to other cells through the release of neurotransmitters. (b) Neurons have axodendritic processes that enable them to send and receive these signals to and from specific targets cells across long distances. While these two properties are combined in modern neurons, they may have evolved independently and at different times (Ryan and Grant 2009). As discussed above, the mechanisms for synaptic signaling, such as various parts of the postsynaptic scaffold, are already present in unicellular organisms and can be said to predate the axodendritic processes that require a multicellular context (ibid.). The question addressed is how protoneurons—defined as cells having (a) but not (b)—capable of synaptic signaling to neighboring cells could have been functional in a way that scaffolded the evolution of full neurons with axodendritic processes, that enabled signaling to non-neighboring cells.

The SBT conjectures that the evolution of the first nervous systems took place in two different phases. The first phase involved the evolution of *excitable* *myoepithelia*. These are epithelia that have both contractile properties (Mackie 1970; Lichtneckert and Reichert 2007) and conduct electrical activity across their surface (Josephson 1985). Such epithelia are both coordinator and effector at the same time. In contrast to modern forms of excitable myoepithelia, the SBT suggests that these early forms were constituted by protoneurons capable of chemical transmission through exocytosis, similar in outline to signaling by chemical synapses (Keijzer et al. 2013).^4^ Such a configuration would consist of an unbroken sheet of protonervous system tissue, each cell signaling to its immediate neighbors. This form of signaling is at heart similar to other forms of chemical signaling between cells and the molecular machinery involved is very old and can have been present at a very early date (Ryan and Grant 2009; Liebeskind et al. 2011). While this form of excitable myoepithelia is hypothetical at present, preliminary modeling studies indicate that such a set up could work as a mechanism for organizing patterned contractions across the epithelium (De Wiljes et al. 2010).

In a second phase, such a protonervous system would evolve a comparatively small number of more specialized signaling cells with *elongated axodendritic processes* by means of which those cells could send signals to nonadjacent cells across the epithelium. This would result in a diffusely connected nerve net, a nervous system that is spread out across the animal body with no or very little cephalization and generally considered as the most primitive form of nervous systems (e.g. Miller 2009; Moroz 2009). There are many possible reasons for initiating this second step, which range from minimizing metabolic costs, enabling more reliable signaling through connections that were not on the surface of the animal, and the ability to cross gaps between non-adjacent contractile tissues.

With the advent of nerve nets and the comparatively long distance connections provided by the neurons constituting them, the placement and form of both contractile surfaces and nervous systems can become much more variable. The contractile apparatus could eventually be cut loose from its placement on the animal surface and repositioned as internal muscle while remaining integrated by the long distance connections of the nerve net. Also, true neurons can themselves be repositioned within the body while their functioning is maintained through long distance connections. Clustering of neurons and cephalization subsequently becomes possible where main parts of a nervous system are repositioned within the animal body and separated from the contractile surface. From here on there is a route of general differentiation leading to complex nervous systems and animal bodies that we are all familiar with and which fits the input–output view so well.

Summarizing, the SBT constitutes a coordination view that outlines a gradual evolutionary change of functional coordinative structures that starts with excitable myoepithelia and progresses to full nerve nets composed of neurons. This skin brain scenario involves a reinterpretation of early nervous functioning that diverges from the idea that nervous systems at a fundamental level are a conductive or information processing system that is only connected to the world through its sensors that provide it with input and effectors that transduce internal processing into events that effect the environment as output. For both these connections, the SBT has a different story to tell and together these lead to the formulation of a specific animal sensorimotor organization that is shared by animals relying on contraction-based motility. The next two sections will develop this idea of a specific ASMO by discussing how the SBT deals with motility and sensing respectively.

## Pantin patterning instead of output

The SBT claims that early nervous systems achieve motility in a way that is not suitably characterized as output. In this context, ‘output’ refers to the idea that a nervous system’s internal operation can be meaningfully dissociated from the behavior or activities that it initiates or controls. The classic notion of ‘motor control’ would provide an example where neural signals initiate and control motility (e.g. Orlovsky et al. 1999). In contrast, skin brains—taken here to include both myoepithelia and diffuse nerve nets—are conceptualized as physically intertwined with a contractile surface instead of constituting a separate controlling system. A second and deeper contrast concerns the foundational role of the contractile surface with respect to a skin brain organization. The latter is not an independently existing controlling system but an organization built in direct connection to a specific contractile tissue across which it helps to modulate electrical and contractile activity. Appreciating this point involves a mind switch away from a general agent-style interpretation of nervous system operation and towards a focus on the requirements imposed by the need to generate patterns of contraction and extension across a specific physical surface.

The starting point for the skin brain proposal is a contractile tissue that extends across a significant portion of the animal body. Patterns of contraction across this tissue will allow the organism to move in specific ways. The total contractile surface that an animal has available for motility will be referred to as an animal’s *Pantin surface* (Keijzer et al. 2013).^5^ For each animal species—and ultimately for each individual animal—this Pantin surface will have a specific size and shape. Muscle-based motility can then be conceptualized as deriving from the systematic patterning of contraction-extension across such a Pantin surface, which will be referred to as *Pantin patterning*. A simple example would be the coordinated contraction of the bell of a jellyfish. The functional result is that it propels water out of the central cavity, pushing the animal forward. Still, what the nervous system does is initiating a synchronized contraction across that particular jellyfish’s Pantin surface.

Thinking in terms of Pantin surfaces and Pantin patterning provides a useful way to generalize across the enormous variety of animal anatomies and to focus on what is common to all cases: Pantin patterning. Obviously, what outward behavior is actually produced will depend on the detailed morphology of individual animals and the environments they act upon, such as in the jellyfish example. However, the shift in perspective involved is that neural activity is not interpreted and described in terms of the perceptual and behavioral functionality that it enables, but as an organ—a tissue organization—that can induce intricate patterns of activation across its associated Pantin surface.

The point can be linked to Maturana and Varela’s (1980) influential differentiation between external organism-environment relations (structural coupling) and the local physiological operation of the organism (operational closure). The operation of nervous systems and the sensorimotor organization itself is not conceptualized in terms of external stimuli or the outwardly visible behavioral tasks that it enables. Instead, an account is provided of what nervous systems and their sensorimotor organizations do in which *not the task or stimulus is held central but the animal itself.* The concept of a behavioral task—e.g. feeding or mating—is not intrinsically tied to any particular kind of organism and assumes an agent that can execute this task (Keijzer 2006). As an analogy, understanding how a digital computer works should be held separate from the content of the specific programs or games that it runs. Similarly, Pantin patterning refers to happenings within particular animal organizations that can, and should be understood in their own right. At the same time, this organization constitutes the means by which functional behavior is achieved.

As a result, the conceptual question how early nervous systems may have functioned moves away from a focus on external circumstances—e.g. to what stimuli does the animal react—and towards understanding how various contractile organizations may have operated and how neural-like features may have enabled or changed various ways of operation. Such patterning is a major and general phenomenon in biology whenever specific biological shapes are produced, for example in animal morphogenesis. A Pantin surface constitutes a so-called excitable medium (Keijzer et al. 2013) across which excitatory and inhibitory activity can spread at various speeds producing large-scale patterns of contraction-extension by means of self-organization (e.g. Ball 2009; Goodwin 1994). How skin brains may specifically have contributed to patterning across this surface is a huge empirical question that is beyond this paper. Important ingredients are likely to be speed of activity spread, excitatory and inhibitory activity, jumping across areas by long-distance neural projections and the specific shape and size of the Pantin surface such that patterning can become harnessed and standardized in a way that is useful for contraction-based motility. The last point provides a key evolutionary link with developmental biology, as Pantin patterning can be presumed to require a standardized body—unlike for example fungi, plants and sponges—and constrained developmental processes that ensure such standardization. This link to developmental processes is likely to extend to the conceptual approaches provided by developmental biology (e.g. Levin 2011; Newman et al. 2006) as Pantin patterning can be seen as just another case of principles that are operating much more widely in animal organization.^6^

Summarizing, Pantin patterning is ill-described as output for various reasons. First, there is no dissociation between a controlling and a controlled system, there is simply a single contractile tissue with neural features incorporated or attached to it. Second, the Pantin surface is a physically circumscribed tissue with a particular size and shape that changes during individual development, growth, aging and damage. Patterning will be particular for that tissue and reflect the animal’s body rather than a generic output function that refers to a generalized functional behavior description, such as feeding or mating. Finally, Pantin patterning always involves a whole behaving organism acting as a unit. Again this diverges from the output notion, which can easily dissociate individual reflexes and stimulus reactions from the animal as a whole. I conclude that the skin brain proposal provides a way to characterize the mechanisms behind animal motility that diverges from the output side of the input–output view.

## Sensing bodies rather than input

Pantin patterning provides the first half of an argument for an interpretation of the animal sensorimotor organization, ASMO, which diverges from the standard input–output view. Is there a second half to this argument or does the SBT still require the notion of ‘input’ to describe sensing as enabled by early skin brains? Sensing and sensitivity are used here according to a reading that Gibson described as “*to detect something*” and contrasted with “*to have a sensation*” (1966, p. 1). Sensing is here not used to refer to mental or intentional aspects of the notion of ‘sensing’. Sensing occurs when a specific environmental structure leads to a change in the organism that allows the organism to behave differently towards this environmental structure because of that change. I will argue that the strict organism-based way of casting this sensitivity by the SBT leads to a form of sensing that is not suitably described as input.

To start, it is important to stress that all living cells are sensitive to a wide variety of external chemical and physical features, which makes every cell capable of acting as some kind of sensor. Cellular sensitivity will also be present in the cells constituting nervous systems (Smith 2008; Greenspan 2007). The sensitivity of single cells can in certain cases be used by multicellular organisms to orient behavior without any nervous system (Leys and Degnan 2001; Nordström et al. 2003). However, the paradigmatic sensory device for animals with nervous systems is a multicellular spatially extended sensor array consisting of many parallel operating sensitive cells, with the retinal and skin surfaces as prominent examples (Gibson 1966). A single-cell sensor can signal the presence or intensity of an environmental feature—such as light—but sensor arrays enable organisms to become sensitive to patterns across extended surface arrays (Gibson 1966; Braitenberg 1984). It cannot be stressed enough how sensitivity to such macroscopic spatiotemporal structure of an environment is essential for the animal way of life. Without it the world of things, places and events that we and other animals inhabit would simply not be accessible. Given their central importance, the question here becomes: How did such sensor arrays and their neural infrastructure first evolve?

In Braitenberg’s illustration of the classic input–output view, sensor arrays derive from the evolutionary accumulation of spatially organized individual sensors into increasingly complex arrays and circuits geared to detect complex environmental features. Interestingly, such sensor arrays rely to a large extent on connections and interactions between cells within the array; that is to connections transverse to the input–output direction. As the importance of such transverse connections is well-established in neuroscience, one of the assets of the skin brain proposal is its focus on the evolution of a transverse nerve net organization spread out across the animal body. Such a nerve net organization provides a good precondition for the evolution of more specific sensor arrays—most notably related to touch—dispersed across the animal body. Given the general sensitivity of individual cells to environmental stimuli, one can envision how a skin brain organization incorporates cellular sensitivity to influence its patterning activity in ways that are generally beneficial for the organism, say in amount of food uptake. The transversely organized skin brain would be well-suited to combine patterns of stimulation across its surface because its main activity relates to organizing such patterning for motility.

This point becomes even stronger as soon one realizes that Precambrian animals must have been soft-bodied and eyeless (e.g. Dzik 2007; Parker 2003), conditions that preclude a stable physical platform for any sensor array. In contrast to vision with a comparatively small retina that can be stabilized, the epithelia of soft-bodied organisms are ongoing contracted and stretched, making a stable and precise configuration of tactile sensor cells challenging. This may seem problematic when it comes to providing stable and reliable input patterns, but these contractions and extensions are mostly self-induced and not random. Therefore, any such epithelial sensor array should not be considered as a passive and static sensor surface but rather as an active exploratory sensor device that actively interacts with its environment. This idea closely fits O’Regan and Noë’s sensorimotor theory of perception (2001; O’Regan 2011). They stress how self-initiated movements cause systematic and specific changes across sensory surfaces—what they call sensorimotor contingencies—to which an animal can become attuned (ibid.). Applied to the present case, endogenously produced Pantin patterning would lead to movements that change the sensor array in specific ways, which in turn would influence the skin brain and its patterning such that it compensates for and adapts to external disturbances of its ongoing activity. Sensing and moving are intrinsically tied together here.

The idea of a deep and intrinsic connection between sensing and moving is now widely established and specifically developed within embodied approaches to cognition (Hurley 2001; Chemero 2009; O’Regan and Noë 2001; O’Regan 2011). Embodied cognition stresses, in various ways, that the coupling between acting and sensing is the key to intelligence and distances itself from the notion of an internally situated ‘intelligent system’. The SBTs stress on the interweaving of contractile and sensory surfaces not only fits in with these embodied approaches but also adds new and more specific ideas concerning the basic organization of the animal sensorimotor organization and the intrinsic connection between moving and sensing here.

Embodied approaches stress the intrinsic connection between sensing and moving but so far did not yet apply this idea to the animal organization itself, taking organisms with extended sensor arrays and definite action capabilities as a starting point. The SBT highlights the problem how early multicellular organizations became sensitive to extended surface structure in the first place (Sheets-Johnstone 2011): What kind of tissue organization could first come to incorporate and use—and in that way sense—external spatial patterns given its own flabby constitution? How can surface structure become meaningful to a biological organization that is initially organized at a cellular and biomolecular level? A particular proximate organization is required that makes this incorporation possible. At this point, the SBT provides a radical idea: *The body itself becomes a sensing device that is at heart independent of input or external sensors*. A skin brain organization provides the means to allow an animal to differentiate between external surface structures in a way that does not build on sensory input as a precondition.

In a nut-shell, the SBT states that skin brains need to be sensitive to the internal Pantin patterns they generate; therefore the resulting embodiment will also become sensitive to environmental structure that impinges on this patterning and changes it. This sensitivity would be solely based on self-initiated movements and internal feedback derived from mechanical obstructions of the animal’s body and does not require sensors triggered by events outside the body. The basic idea is described by O’Shaughnessy (1989) in an intuitive way when he discusses the case of a blind man with a cane who should be able to probe his environment with this cane even without skin contact merely by muscle sense triggered by the resistance encountered with his cane.

Let’s unwrap the idea in some more detail. To induce motility through contraction, specific patterns of contraction-extension fitted to a particular Pantin surface are required to induce, for example, peristaltic movement across a worm’s body. While the Pantin surface is comparatively fixed and stable for any given animal—allowing standardization of patterning for separate species—it is not completely fixed. Various forms of variability at various time-scales are present, ranging from longer-term changes such as growth, short-term changes in stress and activity produced by the activation and movements themselves, and various features such as temperature, chemical changes and also damage and dysfunctions. As such inevitable variability could easily disturb global patterning—making it dysfunctional—skin brains must be sensitive to the ongoing Pantin patterning and counteract disturbances of functional contraction patterns.

To do this, skin brains must modify local cellular and biomolecular features in ways that modify global Pantin patterning such that functional movements are generated. As mentioned above, this requirement is highly similar to morphogenetic and regenerative processes during development where local activity is also dependent on its position within the global organization of the developing organism (Levin 2011, 2013). I will just assume for now that biomolecular and cell-level configurations can accomplish this task, one option being the use of stress-sensitive ion-channels that modify conduction characteristics (Gottlieb and Sachs 2012). In this case, the animal involved will become sensitive to the ongoing dynamical state of its own body: the global bodily distribution of contraction-extension at any particular moment in time. In a way this is a primitive form of proprioception but more basic and without specific muscle-based sensors. Such an arrangement binds the motile body of the animal into a unified dynamic whole that is sensitive to the ongoing dynamical contraction changes across this body and influencing the processes that generate and maintain these same patterns. The activity across—and thus *within*—the skin brain is directly sensitive to and reflecting the spatial lay-out of the animal body. Neural activity is at a deep level attuned to spatiotemporal structure at the bodily scale and intrinsically reflects this spatiotemporal structure. Because of their own activity as patterning devices, skin brains become sensitive to the spatiotemporal dynamics of their own extended bodies.

**Fig. 1:**
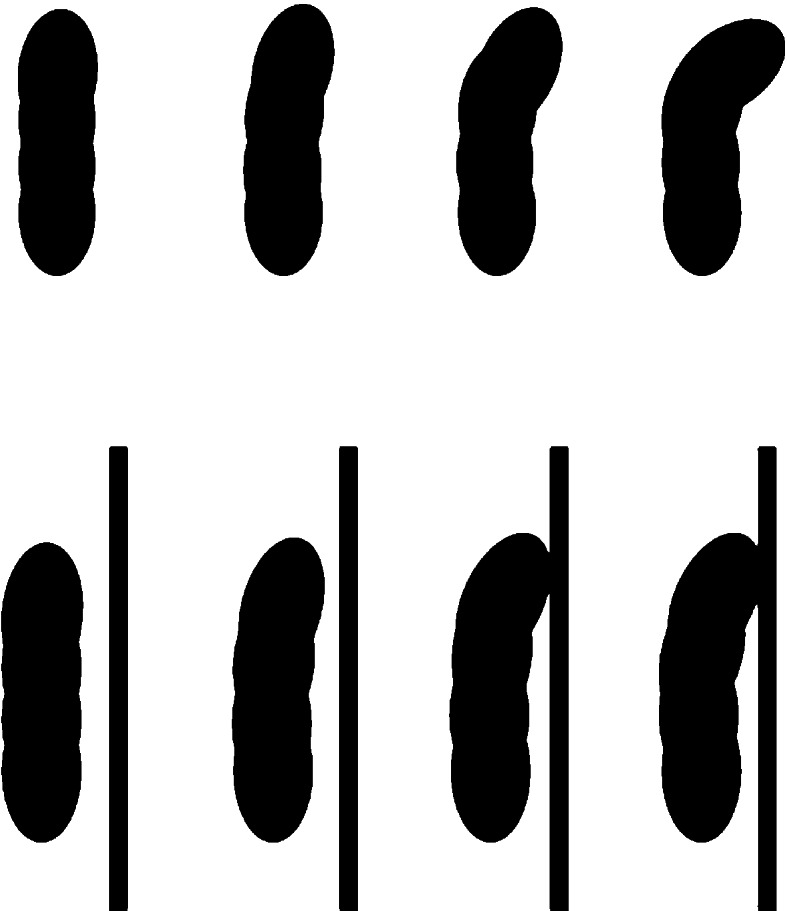
Sensing bodies: a self-induced motion to the right can either proceed (*top*) or is blocked by an external obstacle (*below*). The difference in body contraction can be detected without requiring external sensors

Given that a skin brain organization enables an organism to become sensitive to the dynamics of its own body, such sensitivity could—even must—extend to external surfaces that touch and impinge on this body. A skin brain organization is presumed to be present in simple, soft-bodied organisms, presumably small and crawling while in direct physical contact with the environmental substrate. For such an organism, sensitivity to the bodily dynamics of contraction-extension would also take in external disturbances in Pantin patterning when external surfaces impinge upon that body by either allowing or hindering body contractions or extensions (see Fig. 1). When the external substrate obstructs bodily movement, the animal would sense the obstruction as a disturbance of patterning within the Pantin surface even without *any* dedicated external sensor. Internal sensitivity suffices to generate a basic form of active touch (Gibson 1962; O’Shaughnessy 1989).

The sensor-less sensing body is an *idealization*, aimed to focus on the central idea that a skin brain organization turns the animal body itself into a new kind of multicellular organization that is capable of accessing and handling extended environmental surface structures such as shapes, textures and movements. Skin brains provide an organization that can become differentially sensitive to environmental features at a bodily scale *because* it must be already differentially sensitive to the spatial dynamics across its own Pantin surface. This internal bodily sensitivity provides the scaffold upon which a similar sensitivity to external spatial organization becomes possible.^7^ This direct linkage between internal and external structure provides the foundation of the specific *animal* sensorimotor organization, the ASMO.

The sensor-less case highlights the contrast with the input–output view as it provides a form of sensing without external sensors providing input. At the same time, bodily sensing does not diminish the importance of external sensor arrays. However, instead of watering down the ASMO concept, sensor arrays easily fit within the ASMO and augment it: Sensors do here not function as input devices as specified by the input–output view. Bodily sensing provides a fundamental example of systematic relations between self-initiated motion and feedback. Initially from the contractile surface itself, but from here on, the same principle can be used when motion-induced changes across external—or internal—sensor arrays feed back into nervous system activity resulting in various sensorimotor contingencies. Which contingencies would depend on the kind of energy the sensor arrays are sensitive to—e.g. pressure or electromagnetic waves. But such differences become superficial compared to the fact that the same principle of various sensorimotor contingencies applies to all, providing different ways of sensing that enable animals to orient themselves with respect to environmental surface structure.

Adding external sensors to the ASMO therefore does not imply a return to the input–output view. It actually strengthens the notion of an ASMO and makes it more versatile. An interesting implication is that while O’Regan and Noë’s original formulation of the sensorimotor theory focused on human vision and assumed the presence of sensors and effectors, the SBT provides the outlines of a foundation of a sensorimotor theory at a very early stage of nervous systems evolution, while making sensorimotor contingencies a crucial feature of animal sensing.

To summarize, sensing as accomplished by animals with skin brains is insufficiently described as ‘input’. Instead, the animal body itself becomes a sensing device through its use of contractile tissues and the environmental feedback this generates, both within and external to the body. Together, this constitutes a specific *animal* sensorimotor organization, or ASMO, that differs from the input–output view on both nervous system and the sensorimotor architecture of animals.

## Concluding: the animal sensorimotor organization (ASMO)

This paper aims to address a seemingly fundamental limitation of a specific coordination view on the origins of nervous systems: As the SBT emphasizes the evolutionary need to coordinate motility it seems to ignore the sensory aspects of early nervous systems. In response, I sketched how the SBT provides an account of moving *and sensing*. Instead of neglecting sensory issues, this coordination view provides a new account of sensing as enabled by early nervous systems: The skin brain organization provides a specific *animal* sensorimotor organization that differs from the input–output view’s interpretation of a sensorimotor organization.

A good way to highlight the differences in some detail is by returning to the eight typical features of the input–output view, derived from Braitenberg’s vehicles in “The input–output view on nervous systems and the animal sensorimotor organization” section, and to contrast them to their ASMO counterparts:

1. The ASMO operates in a way that is different from current artificial sensorimotor organizations;
2. multicellular sensors and effectors are not given but derived from nervous systems;
3. a diffuse nerve net is the most basic form of a nervous system;
4. the ASMO is necessarily active, motility is a precondition for sensing;
5. activity within early nervous systems is primarily transverse to sensor-effector connections and spread out across sensor and effector surfaces;
6. nervous systems depend on their own generic patterning processes;
7. soft animal bodies are instable platforms for sensors and effectors, sensing and motility are actively stabilized by relying on sensorimotor contingencies;
8. the animal’s body and nervous system are fully integrated.

Given this point for point comparison, it becomes evident that the SBT and the ASMO provide a major conceptual reinterpretation of how early animals moved and sensed, compared to the input–output view. This reinterpretation also fits in with and contributes to current work in the cognitive sciences that stresses the importance of embodiment and sensorimotor relations for intelligence. The ASMO provides a new view on embodiment and sensorimotor organization, adding to ongoing work on the embodied aspects of cognition that will be important to pursue for more complex cases, such as recently proposed by Turvey and Fonseca (2014).^8^

The overall argumentative aim of the paper was to add to the available scenarios for early nervous system evolution by elaborating a particular coordination view on these events. Highlighting the differences with an input–output is not to criticize the latter but to clarify this coordination view. The major aim was to establish that the SBT significantly widens our conceptual options in dealing with early nervous systems: it provides new ideas, new questions and new suggestions for research. In a field as difficult as the evolution of early nervous systems, where so little is actually known and where conjecture still reigns, providing more clear and specific ideas on what could have happened must be considered progress.

## Footnotes

1 Reviews of this literature can be found in Mackie (1990), Lichtneckert and Reichert (2007) and Moroz (2009, 2014), among others. A short overview of the issues can be found in Miller (2009).

2 The phrase ‘skin brain’ is derived from Nicholas Holland (2003), but is here used in a way that diverges from the particular interpretation he gave to it.

3 Nervous systems do obviously much more than controlling contraction, such as controlling secretory organs and many metabolic functions. This is not denied, nor is it implied that this is not essential (Jékely et al. in preparation). The present text explores the conceptual option that controlling contraction-based motility was the primary driving force behind the early evolution of nervous systems.

4 Excitable myoepithelia occur in modern animals such as jellyfish, where epithelial cells are directly electrically coupled through gap junctions. However, molecular evidence suggests that gap junctions evolved later than nervous systems (Satterlie 2011), making the excitable myoepihelia as proposed here a plausible precursor.

5 A Pantin surface as discussed here is taken to consist of a single continuous muscle sheet, such as an excitable myoepithelium. In more complex cases, involving more extensive neural connections, a Pantin surface could become dispersed across various muscle groups.

6 While the present focus is on very early nervous systems, the notion of patterning is an intriguing way to address the wide variety of anatomical, molecular and physiological features of nervous systems that are difficult to relate to standard information processing accounts (e.g. Bell 1999; Bullock et al. 2005; Parker 2010; Raichle 2010).

7 An important background assumption that is not discussed here consists of the metabolic and energetic requirements of living organizations (Rosslenbroich 2014). Obviously, the SBT presented here will have to link up with these core biological requirements at a cellular and biomolecular level (e.g. Barandiaran and Moreno 2006; Thompson 2007). Current work on the stress response (Lyon 2013) is an important direction for developing this link in more detail.

8 I want to thank Martin Fultot for bringing this connection to my attention.
